# Plant-exclusive domain of *trans*-editing enzyme ProXp-ala confers dimerization and enhanced tRNA binding

**DOI:** 10.1016/j.jbc.2022.102255

**Published:** 2022-07-12

**Authors:** Jun-Kyu Byun, John A. Vu, Siou-Luan He, Jyan-Chyun Jang, Karin Musier-Forsyth

**Affiliations:** 1 Center for RNA Biology, The Ohio State University, Columbus, Ohio, USA; 2 Department of Chemistry and Biochemistry, The Ohio State University, Columbus, Ohio, USA; 3 Department of Horticulture and Crop Science and Center for Applied Plant Sciences, The Ohio State University, Columbus, Ohio, USA

**Keywords:** aminoacyl-tRNA, aminoacyl-tRNA synthetase, *Arabidopsis thaliana*, bimolecular fluorescence complementation, enzyme kinetics, molecular modeling, plant biochemistry, translational quality control in plants, *trans*-editing domains, aa-tRNA, aminoacyl-tRNA, aaRS, aminoacyl-tRNA synthetase, At/A. thaliana, Arabidopsis thaliana, BiFC, bimolecular fluorescence complementation, CTD, C-terminal domain, ΔCTD ProXp-ala, At ProXp-ala with truncated CTD, ES, enzyme–substrate, FL, full length, INS, insertion, MSC, multi-aminoacyl-tRNA synthetase complex, ProRS, prolyl-tRNA synthetase, SEC-MALS, size-exclusion chromatography with multiangle laser-light scattering, STO, single turnover

## Abstract

Faithful translation of the genetic code is critical for the viability of all living organisms. The *trans*-editing enzyme ProXp-ala prevents Pro to Ala mutations during translation by hydrolyzing misacylated Ala-tRNA^Pro^ that has been synthesized by prolyl-tRNA synthetase. Plant ProXp-ala sequences contain a conserved C-terminal domain (CTD) that is absent in other organisms; the origin, structure, and function of this extra domain are unknown. To characterize the plant-specific CTD, we performed bioinformatics and computational analyses that provided a model consistent with a conserved α-helical structure. We also expressed and purified wildtype *Arabidopsis thaliana* (*At*) ProXp-ala in *Escherichia coli*, as well as variants lacking the CTD or containing only the CTD. Circular dichroism spectroscopy confirmed a loss of α-helical signal intensity upon CTD truncation. Size-exclusion chromatography with multiangle laser-light scattering revealed that wildtype *At* ProXp-ala was primarily dimeric and CTD truncation abolished dimerization *in vitro*. Furthermore, bimolecular fluorescence complementation assays in *At* protoplasts support a role for the CTD in homodimerization *in vivo*. The deacylation rate of Ala-tRNA^Pro^ by *At* ProXp-ala was also significantly reduced in the absence of the CTD, and kinetic assays indicated that the reduction in activity is primarily due to a tRNA binding defect. Overall, these results broaden our understanding of eukaryotic translational fidelity in the plant kingdom. Our study reveals that the plant-specific CTD plays a significant role in substrate binding and canonical editing function. Through its ability to facilitate protein–protein interactions, we propose the CTD may also provide expanded functional potential for *trans*-editing enzymes in plants.

Aminoacyl-tRNA synthetases (aaRSs) are universally conserved enzymes that ensure high fidelity of translation of genetic information into functional proteins across all domains of life. These enzymes pair amino acids with their corresponding tRNA isoacceptors in a process known as aminoacylation, which takes place in two steps: amino acid activation and transfer to tRNA. First, aaRSs use ATP to activate amino acids, forming an aminoacyl-adenylate intermediate. Next, the activated amino acid is transferred to either the 2′- or 3′-hydroxyl group of the terminal adenosine of the tRNA acceptor stem to form aminoacyl-tRNA (aa-tRNA). The aa-tRNA is delivered to the ribosome by elongation factors to take part in protein synthesis (1). While correct tRNA selection by aaRSs is facilitated by the large tRNA surface area available for recognition, the size and functional group similarity among related amino acids make it challenging for aaRSs to accurately distinguish these smaller substrates (2, 3, 4, 5, 6). Incorporation of incorrect amino acids into the nascent polypeptide chain during translation can lead to inactive or misfolded proteins. Accumulation of these aberrant proteins can cause diverse cellular and organismal defects ranging from cell death in microbes to neurodegeneration in mammals (7, 8, 9, 10, 11, 12).

Despite the challenges in amino acid selection by aaRSs, errors in translation only occur about every 1 in 10,000 codons; this suggests the existence of proofreading mechanisms to edit the majority of aa-tRNA mischarging events prior to translation at the ribosome (13, 14, 15, 16). Indeed, many aaRSs have acquired two distinct editing mechanisms, termed pre- and posttransfer editing, that prevent formation and/or accumulation of mispaired tRNAs (6). In pretransfer editing, the aminoacyl-adenylate is hydrolyzed prior to the transfer step, and in posttransfer editing, the ester linkage of the mischarged aa-tRNA is cleaved, releasing the tRNA and free amino acid. This type of editing can occur in *cis*, wherein the mischarged aa-tRNA is translocated from the aaRS aminoacylation active site to a distinct editing domain where deacylation occurs (17). Alternatively, posttransfer editing can also occur in *trans*, wherein the mischarged aa-tRNA is released and rebound by either an aaRS or a free-standing editing domain for deacylation (18). Free-standing *trans*-editing domains are structurally homologous to editing domains encoded in some aaRSs and have been identified in all domains of life (17).

It is well established that prolyl-tRNA synthetase (ProRS) mischarges Ala and Cys onto tRNA^Pro^; these amino acids are smaller or similar in size to cognate Pro (19, 20, 21, 22). Most bacteria encode a ProRS with an editing domain known as the insertion (INS) domain, which hydrolyzes misacylated Ala-tRNA^Pro^ but not Cys-tRNA^Pro^ (19). The latter is edited by YbaK, a *trans*-editing domain homologous to the INS domain (23, 24). Some bacterial species, such as *Caulobacter crescentus*, possess a ProRS that lacks an INS domain and instead encode a free-standing single-domain protein, ProXp-ala, which is structurally homologous to the INS domain and serves to deacylate Ala-tRNA^Pro^ in *trans* (25, 26, 27). In eukaryotes, including humans, all ProRSs lack an INS domain, so these organisms rely exclusively on *trans*-editing activity to clear mischarged tRNA^Pro^ (26, 28). Recent bioinformatics analyses revealed a superfamily of putative INS-like editing domains including INS, ProXp-ala, YbaK, ProXp-ST1, ProXp-ST2, ProXp-X, and three uncharacterized ProXp domains (17, 26). With the exception of human ProXp-ala, the subset of INS superfamily members that have been characterized *in vitro* and *in vivo* to date has been exclusive to prokaryotic systems (17, 29).

Relatively little is known about translational fidelity mechanisms in nonhuman eukaryotes, especially plants where misincorporation of proteinogenic as well as nonproteinogenic amino acids may be problematic (30, 31, 32). A genome-wide search of aaRS genes in the model plant organism *Arabidopsis thaliana* (*A. thaliana*, *At*) revealed that some aaRSs contain additional domains appended to catalytic domains, while others lack catalytic domains or other portions of the full-length aaRS (33). This survey also identified unique domains found only in plant aaRSs, which are candidates for facilitating canonical or noncanonical roles (33). Our sequence analyses showed that the majority of plant species encode ProXp-ala, and multiple sequence alignments revealed that all plant ProXp-ala proteins contain a conserved C-terminal domain (CTD) of unknown function that is absent from other species. Whether this “extra” domain facilitates canonical function and/or confers a new function to plant ProXp-ala is an open question.

In this study, we investigated the *trans*-editing enzyme ProXp-ala from *A. thaliana* (At1g44835) through both *in vitro* and *in vivo* approaches. *At* ProXp-ala contains a plant-exclusive CTD appended to the N-terminal catalytic domain. We performed computational, biophysical, and kinetic analyses using wildtype (WT) ProXp-ala, as well as a CTD-deletion variant (ΔCTD) and a CTD-only variant. *In vitro* results showed that the CTD contributed to protein homodimerization, Ala-tRNA^Pro^ deacylation, and Ala-tRNA^Pro^ binding of *At* ProXp-ala. To further understand the roles of *At* ProXp-ala in plants, *in vivo* analyses were conducted using *At* mesophyll protoplasts. Results of protein subcellular localization studies indicated that *At* ProXp-ala localized to both the cytoplasm and nucleus. Bimolecular fluorescence complementation (BiFC) assay results showed that the CTD plays a role in homodimerization of *At* ProXp-ala *in vivo*. These data support the canonical function of *At* ProXp-ala in translational quality control and reveal a novel role for the unique CTD in homodimerization and enhanced tRNA binding.

## Results

### Sequence-based analyses and computational structural predictions of *At* ProXp-ala

**Figure 1:**
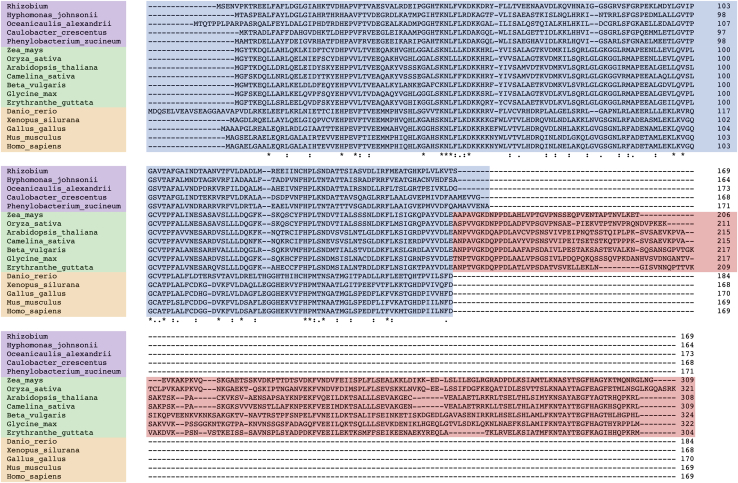
**Multiple sequence alignment of ProXp-ala.** Sequences of representative bacterial (*top*/*purple*), plant (*middle*/*green*), and animal (*bottom*/*orange*) ProXp-ala. The plant-exclusive C-terminal domain is shaded in *red*. Asterisks (∗) indicate positions with strictly conserved residues, colons (:) indicate positions with conserved residues with strongly similar properties, and periods (.) indicate positions with conserved residues with weakly similar properties.

Based on previous bioinformatic analyses, eukaryotic ProXp-ala is widely distributed in vertebrate animals, almost all plant species, and some protists (29, 34). The predicted protein product of the plant ProXp-ala gene is nearly twice the size of both prokaryotic and nonplant eukaryotic sequences due to a unique CTD that shares no primary sequence homology with any protein domain known to be involved in translational fidelity (Fig. 1). Sequence alignment of ProXp-ala from 35 plant species revealed that 61% (101/165) of the plant ProXp-ala N-terminal catalytic domain residues display sequence similarity, with 36% (59/165) of the residues being strictly conserved. However, only 17% (25/143) of residues in the plant CTD display sequence similarity across all 35 species, with 11% (16/143) of residues being strictly conserved (Fig. S1). To reveal the possible origin of the CTD in plants, an extensive BLAST search was performed using the CTD sequence alone. Two hits matched ProXp-ala CTDs in higher plants, whereas, interestingly, a member of the *At* pectin methylesterase inhibitor family (At1g62760, AtPMEI10) shared 41% similarity (58/143) and 25% identity (36/143) with the *At* ProXp-ala CTD; a green algae putative chloroplast-specific DNA endonuclease (YP_76,436) also shared 44% (63/143) similarity and 22% (31/143) identity with the *At* ProXp-ala CTD.

**Figure 2:**
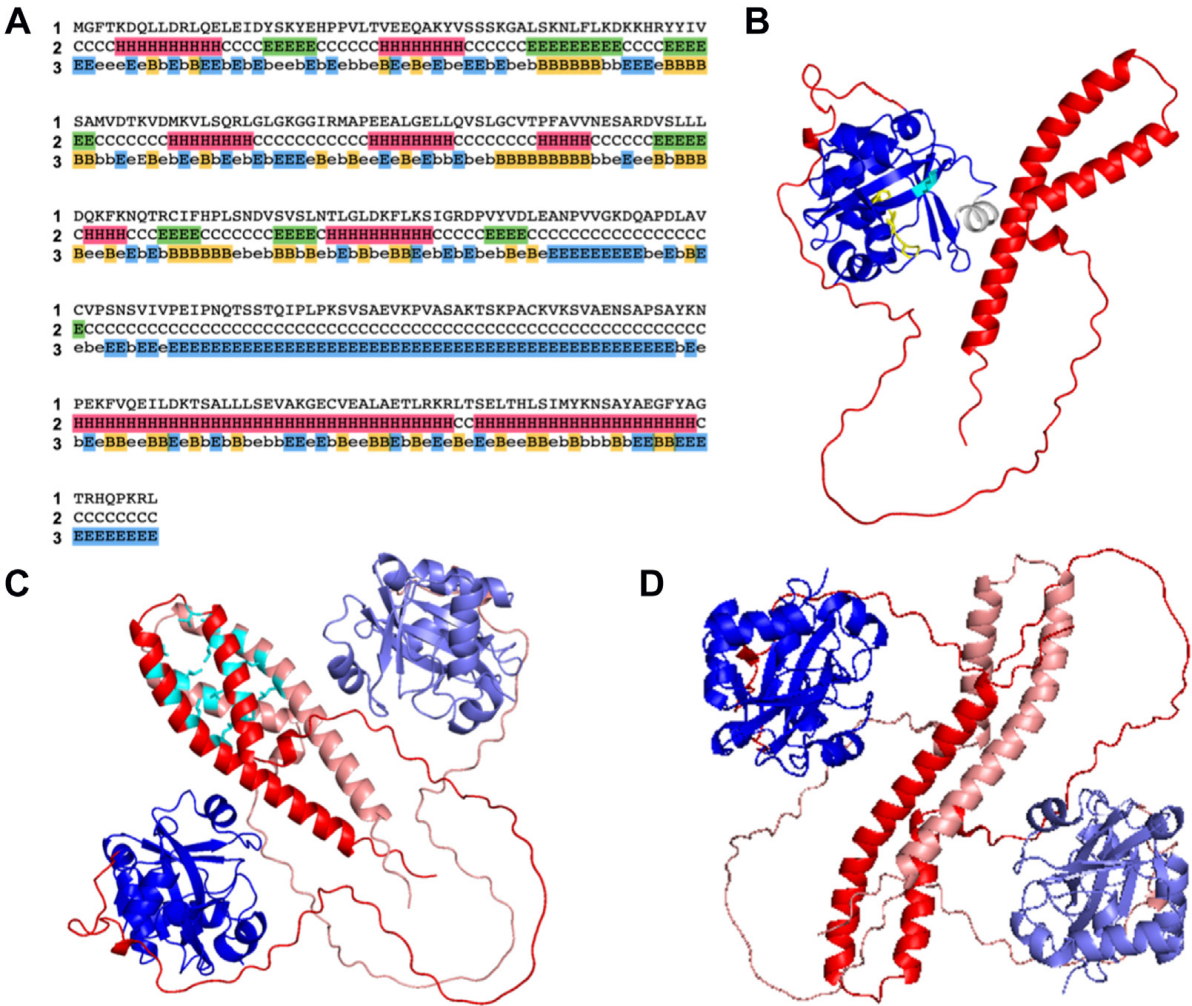
**Prediction of protein structure and dimerization of *At* ProXp-ala.***A*, (1) Primary amino acid sequence of *At* ProXp-ala, (2) Porter 4.0 prediction of secondary structure, and (3) PaleAle 4.0 prediction of relative solvent accessibility. Each residue is predicted to fold into an α-helix (*pink H*), a β-sheet (*green E*), or a random coil (*C*), and be completely buried (*yellow B*), partly buried (b), partly exposed (e), or completely exposed (*blue E*). *B*, AlphaFold protein structure prediction of *At* ProXp-ala showing the conserved N-terminal ProXp-ala catalytic core (*blue*) connected to the predicted alpha-helical domain (*red*) *via* a disordered C-terminal random coil (*red*). The α-2 helix (*gray*), catalytic lysine K46 (*cyan*), and GXXXP loop (*yellow*) that are conserved among *trans*-editing domains are also shown. (*C* and *D*) GalaxyWEB GalaxyHomomer *ab initio* homodimer prediction with parallel CTD interaction (*C*) and antiparallel CTD interaction (*D*). The ProXp-ala and CTD domains of one subunit are shown in blue and red, respectively. The analogous domains of subunit two are shown in *pale blue* and *pale red*.

Computational structural predictions were next carried out for plant ProXp-ala domains. Predictions of the secondary structure and relative solvent accessibility of *At* ProXp-ala, as well as all 34 other plant ProXp-ala sequences using Porter, PaleAle 4.0, indicated that the N-terminal catalytic core contains a pattern of secondary structures consistent with ProXp-ala in other domains of life followed by a random coil linker region that connects to a primarily α-helical C terminus (Fig. 2*A* and data not shown) (35). The *At* ProXp-ala structure predicted by AlphaFold supports a canonically folded N-terminal domain connected to a C-terminal α-helical region *via* a random coil linker (Fig. 2*B*). A similar result was obtained using AlphaFold to predict the structures of other representative plant ProXp-ala sequences (*Zea mays*, *Oryza sativa*, and *Glycine max*) (data not shown) (36, 37).

Homology modeling of the *At* ProXp-ala CTD using SWISS-MODEL revealed a region (Y238-E295) possessing 21% (12/58) amino acid identity with a region (G75-N132) of the *Chlamydia tracomatis* (*Ct*) Pgp3 antigen (38). This region of the solved crystal structure of *Ct* Pgp3 (PDB ID: 4JDM.1.A) is highly α-helical and part of a trimerization interface (39). To probe whether the corresponding region of the *At* ProXp-ala CTD may confer oligomerization potential, the GalaxyWEB GalaxyHomomer *ab initio* homo-oligomer prediction tool was used (40). Based on this analysis, the α-helical region of the CTD was predicted to dimerize in either a parallel or antiparallel manner (Fig. 2, *C* and *D*). Similar residues are positioned to participate in both the parallel and antiparallel homodimer interactions, and they possess primarily β-branched, hydrophobic side chains spaced three to four residues apart.

### The CTD of at ProXp-ala is an α-helical oligomerization domain

**Figure 3:**
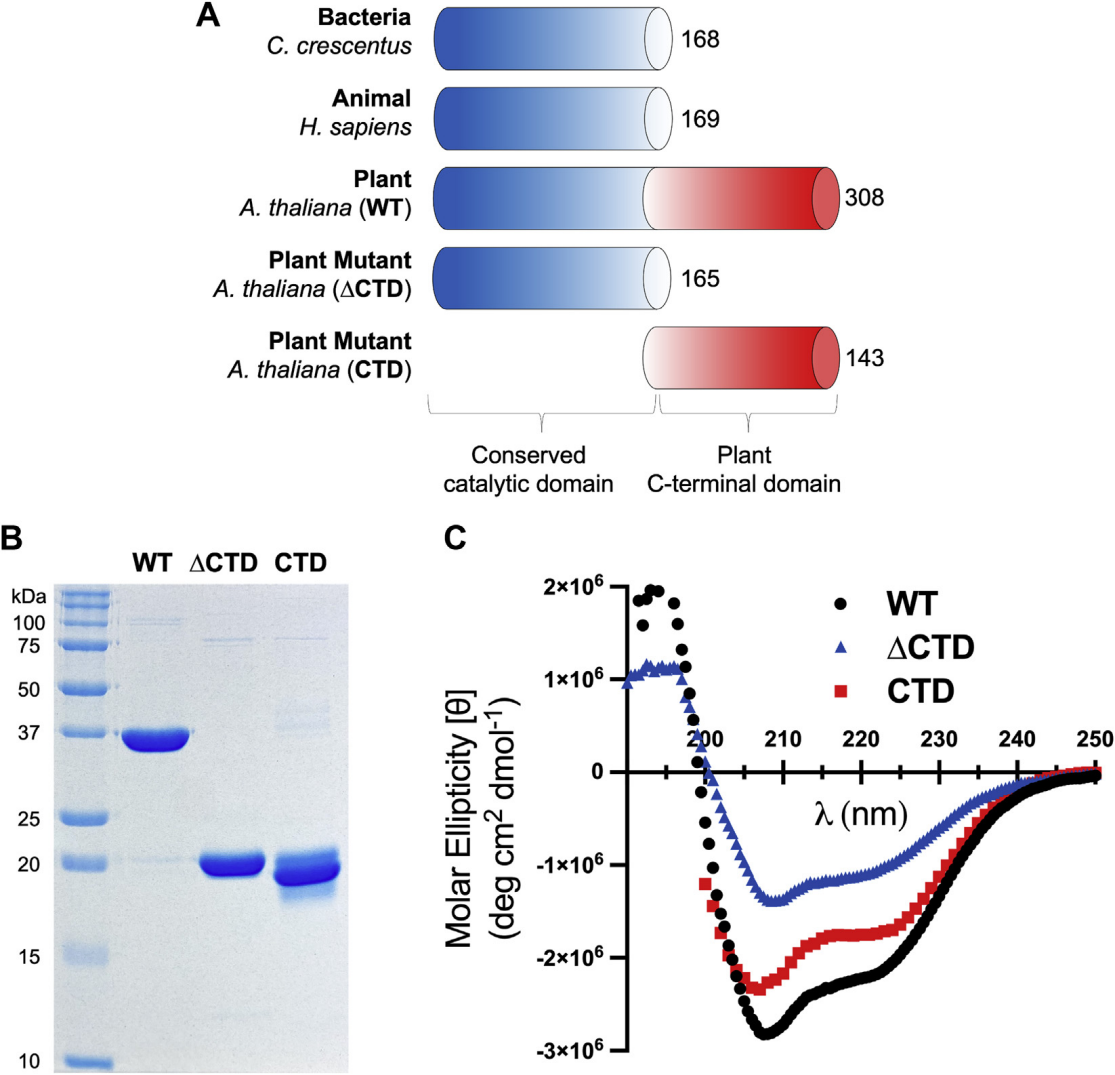
**Domain architecture, purification, and secondary structure of WT *At* ProXp-ala and variants.***A*, domain architecture of bacterial, animal, and plant ProXp-ala (WT) with amino acid counts at the C termini. Two plant ProXp-ala variants used in this study are also shown (ΔCTD and CTD only). *B*, SDS-polyacrylamide (15%) gel of recombinantly purified WT, ΔCTD, and CTD *At* ProXp-ala stained with Coomassie Brilliant Blue G-250. *C*, circular dichroism (CD) spectra of WT, ΔCTD, and CTD *At* ProXp-ala. Spectra were obtained in triplicate and the average is shown, with the exception of the CTD, which is a single trial.

To experimentally characterize the structure of *At* ProXp-ala, recombinant WT *At* ProXp-ala was overexpressed and purified in *Escherichia coli* (Fig. 3, *A* and *B*). The ΔCTD variant lacking the disordered linker and α-helical region and the CTD variant containing only these regions were also overexpressed and purified in *E. coli* (Fig. 3, *A* and *B*). Circular dichroism (CD) spectroscopy experiments shown in Figure 3*C* revealed that WT ProXp-ala displays minima at 208 nm and 222 nm, characteristic of proteins with significant α-helical character (41). Truncation of the CTD resulted in a decrease in α-helical CD signal intensity compared with WT. The CTD alone displayed a CD spectrum characteristic of primarily α-helical structure as well.

**Figure 4:**
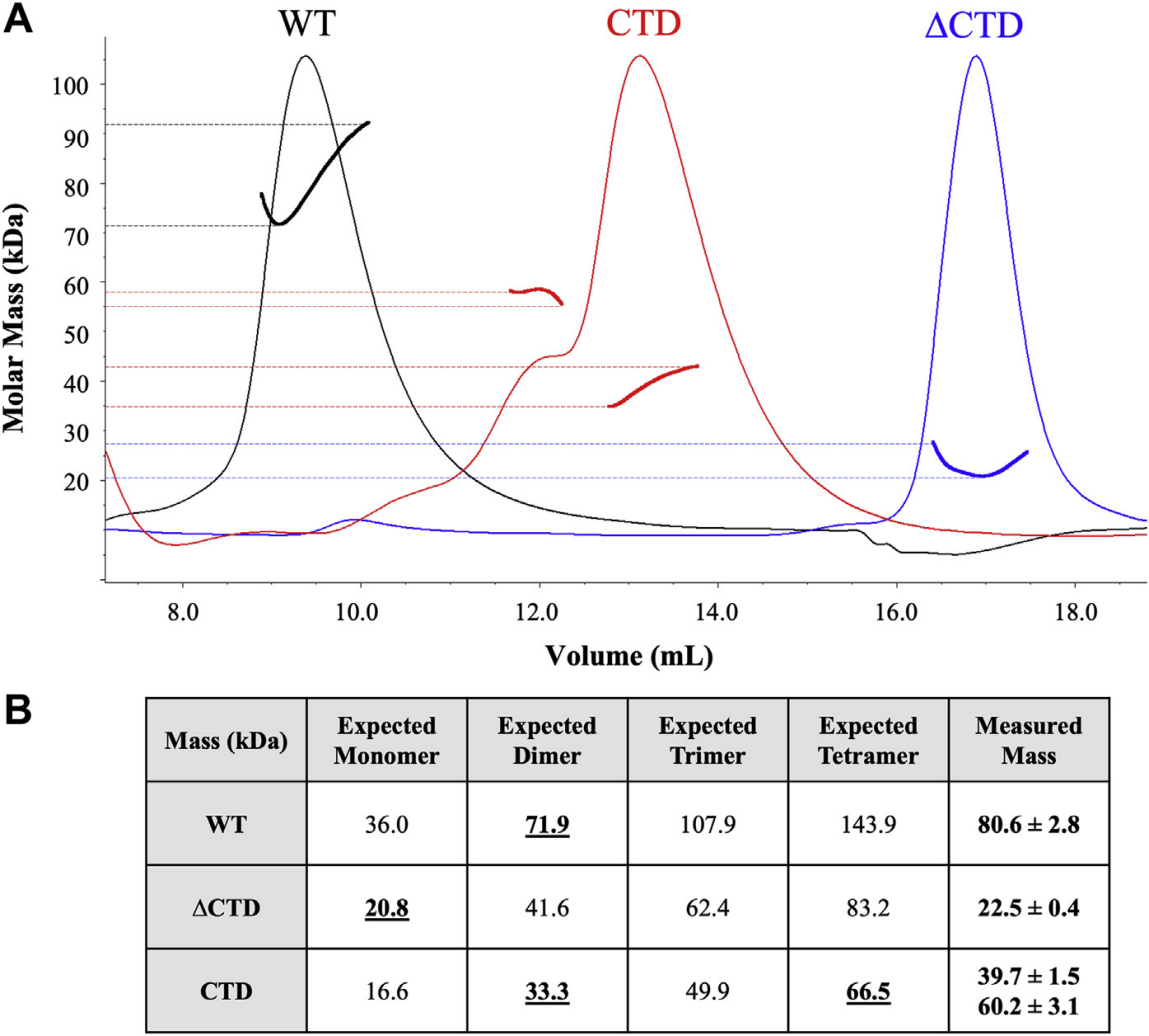
**Characterization of oligomeric state of WT *At* ProXp-ala and variants.***A*, representative size-exclusion chromatography with multiangle laser-light scattering analysis showing light scattering peaks for WT (*black*), ΔCTD (*blue*), and CTD (*red*) *At* ProXp-ala. Individually normalized refractive index (RI) chromatograms are plotted, and *thick lines* represent the calculated molar mass range of particles that eluted within each peak. *Dashed lines* flank the range of masses that are represented by each peak. *B*, tabulated theoretical molecular weights for different oligomeric states and experimental values determined by size-exclusion chromatography with multiangle laser-light scattering. Values are the average of three independent experiments with the standard deviation indicated. Theoretical values that are the most consistent with experimental results are underlined.

To investigate the potential role of the CTD in oligomerization, size-exclusion chromatography with multi-angle laser-light scattering (SEC-MALS) was performed using recombinant purified WT, ΔCTD, and CTD *At* ProXp-ala. The light scattering spectrum of each protein is shown in Figure 4*A*. The measured molecular mass of each protein was determined and tabulated along with the theoretical molecular mass of various oligomeric states (Fig. 4*B*). For WT *At* ProXp-ala, we obtained a molecular mass of 80.6 ± 2.8 kDa, which corresponds closely to the expected mass of a dimer (71.9 kDa). When the same experiment was performed using ΔCTD *At* ProXp-ala, a molecular mass of 22.5 ± 0.4 kDa was obtained, consistent with a monomer (20.8 kDa). Experiments with CTD *At* ProXp-ala yielded two peaks corresponding to molecular masses of 39.7 and 60.2 kDa, which most closely correspond to a dimer (33.3 kDa) and tetramer (66.5 kDa), respectively. These results are consistent with a role for the CTD in protein oligomerization.

### *At* ProXp-ala deacylation of Ala-tRNA^Pro^ is facilitated by the CTD

**Figure 5:**
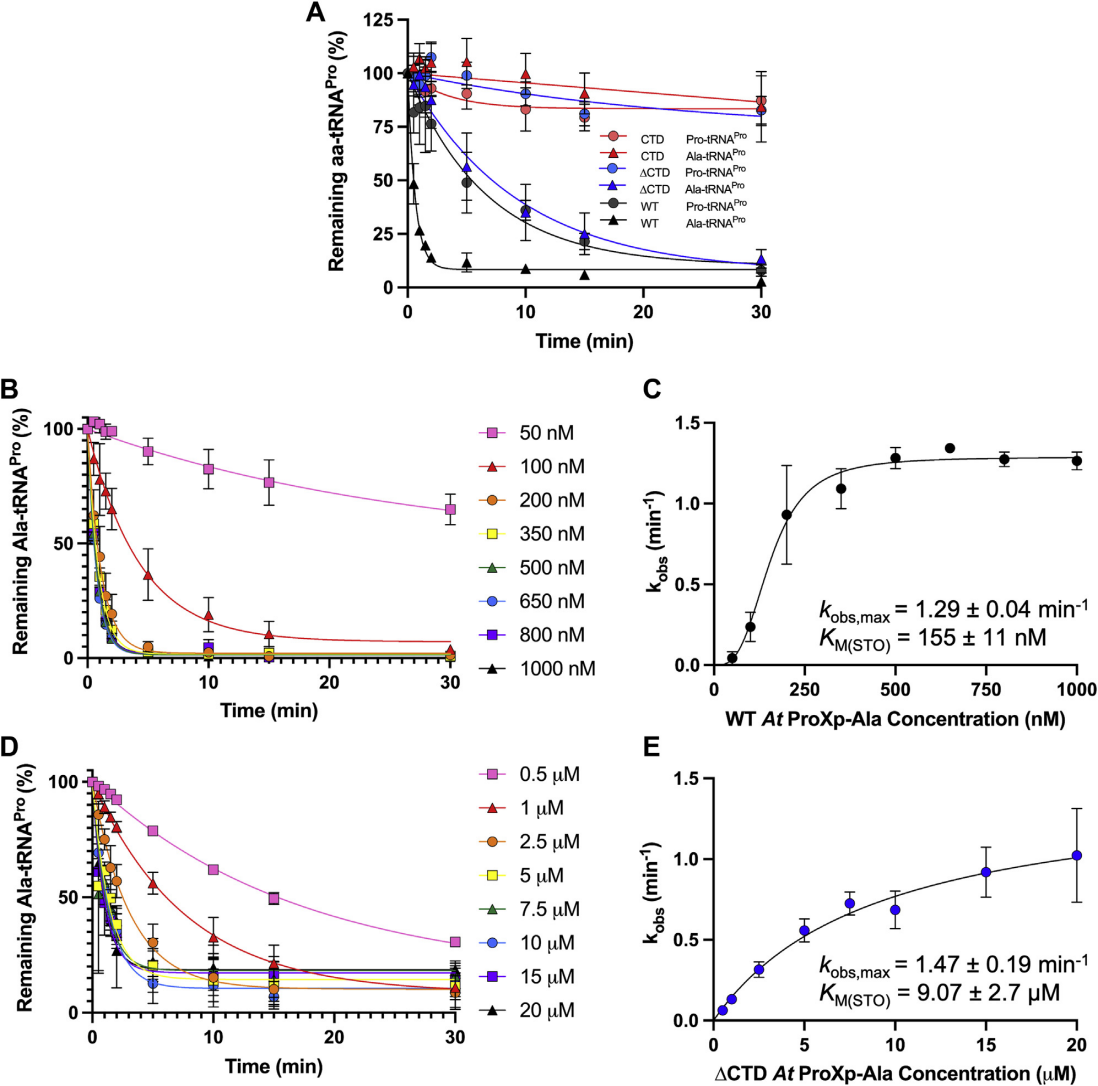
**Deacylation of *At* aminoacyl-tRNAs by *At* ProXp-ala variants.** All reactions were performed under single-turnover conditions with 10 nM aminoacyl-tRNA at 25 °C as described in the Experimental procedures. *A*, Deacylation of *At* Ala- and Pro-tRNA^Pro^ by 500 nM WT, ΔCTD, or CTD *At* ProXp-ala. *B*, time course of *At* Ala-tRNA^Pro^ deacylation with varying concentration (50–1000 nM) of WT *At* ProXp-ala. *C*, sigmoidal fit of *k*_obs_ versus WT *At* ProXp-ala concentration. *D*, time course of *At* Ala-tRNA^Pro^ deacylation with varying concentrations (0.5–20 μM) of ΔCTD *At* ProXp-ala. *E*, hyperbolic fit of *k*_obs_*versus* ΔCTD *At* ProXp-ala concentration. All deacylation curves were background subtracted and fit to a single exponential equation. Each curve is the average of at least three independent trials with standard deviations indicated.

The results of single-turnover (STO) deacylation assays of Ala-tRNA^Pro^ and Pro-tRNA^Pro^ by WT, ΔCTD, and CTD *At* ProXp-ala are shown in Figure 5*A*. Observed rate constants, *k*_obs_, were calculated for each combination of protein and aminoacyl-tRNA^Pro^. WT ProXp-ala exhibited robust deacylation of Ala-tRNA^Pro^, while deletion of the CTD resulted in a 14-fold reduction in deacylation activity under identical conditions. CTD *At* ProXp-ala lacks the catalytic residues for deacylation and failed to display any significant deacylation activity, as expected. WT ProXp-ala also deacylated Pro-tRNA^Pro^, albeit at a 10-fold reduced rate relative to Ala-tRNA^Pro^. ΔCTD ProXp-ala and the CTD alone failed to deacylate cognate Pro-tRNA^Pro^.

To understand whether the reduced Ala-tRNA^Pro^ deacylation activity upon CTD deletion was due to a binding or catalytic defect, we performed STO deacylation assays with varying concentrations of WT and ΔCTD *At* ProXp-ala (Fig. 5, *B* and *D*). Based on these data, we determined the maximum observed rate constant, *k*_obs,max_, and the STO Michaelis constant, *K*_M(STO)_, for each protein (Fig. 5, *C* and *E*). For the WT enzyme, we determined a *k*_obs,max_ of 1.29 ± 0.04 min^-1^ and a *K*_M(STO)_ of 155 ± 11 nM (Fig. 5*C*). For ΔCTD ProXp-ala, *k*_obs,max_ was found to be 1.47 ± 0.19 min^-1^ and *K*_M(STO)_ was 9.07 ± 2.7 μM (Fig. 5*E*). These data indicate that truncation results in no significant change in *k*_obs,max_ and a 59-fold increase in *K*_M(STO)_.

### The CTD of At ProXp-ala enhances Ala-tRNA^Pro^ deacylation by contributing to tRNA binding

**Figure 6:**
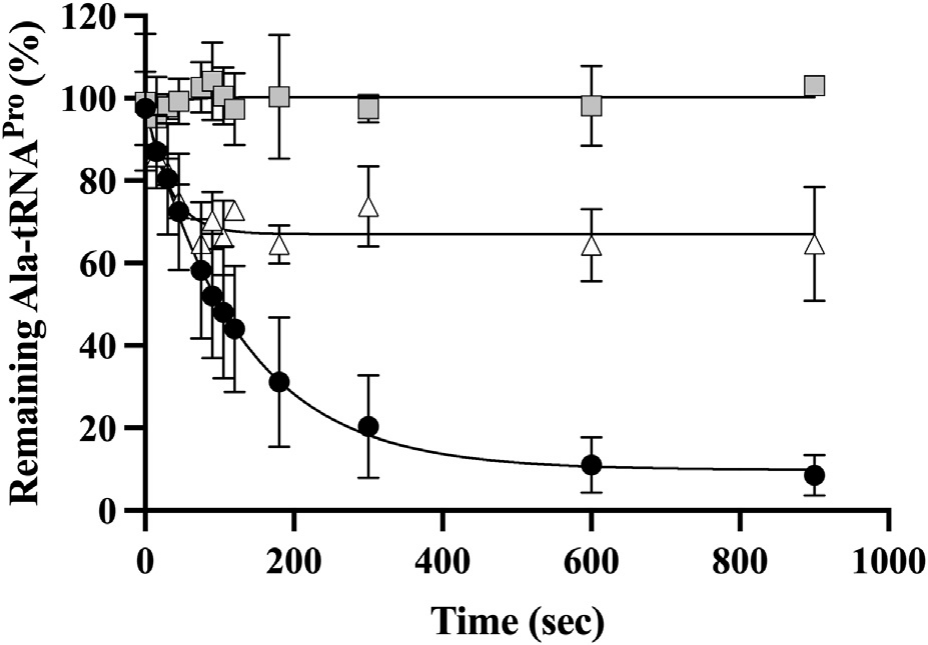
**Pulse-chase experiment with WT *At* ProXp-ala and *At* Ala-tRNA**^**Pro**^**.** Deacylation of 100 nM WT *At* Ala-tRNA^Pro^ by 750 nM WT *At* ProXp-ala was measured without dilution (•) and after 100-fold dilution by 1.0 μM unlabeled, uncharged WT *At* tRNA^Pro^ at 0 s () or 60 s (). Deacylation curves represent the average of three independent trails fit to a single exponential equation with the standard deviation indicated.

Under STO conditions, *k*_obs_ depends on either the binding or chemical steps based on the relative magnitudes of the rate constants of binding, *k*_on_, dissociation, *k*_off_, and catalysis, *k*_cat_. If the enzyme–substrate (ES) complex favors substrate release more strongly than product formation (*k*_off_ >> *k*_cat_), *K*_M(STO)_ corresponds to *K*_d_ and *k*_obs,max_ is equal to *k*_cat_. To determine the relationship between substrate binding and catalysis, a pulse-chase experiment was performed (Fig. 6). In this experiment, a deacylation assay was performed with WT ProXp-ala and radiolabeled Ala-tRNA^Pro^. Following a 60-s incubation to allow for all substrate to bind to enzyme and form ES complexes, the reaction was pulsed with a 100-fold buffer dilution including excess cold tRNA^Pro^. The preformed ES complexes may then either dissociate or form product for the remainder of the reaction. The lack of observable product formation after dilution (Fig. 6) indicates that ES complex dissociation occurs more rapidly than product formation, providing evidence that *k*_off_ >> *k*_cat_. We conclude that *K*_M(STO)_ approximates *K*_d_ and *k*_obs,max_ approximates *k*_cat_. Thus, the observed ∼60-fold reduction in enzymatic efficiency of ΔCTD relative to WT ProXp-ala is primarily due to a substrate binding defect.

### Subcellular localization of ProXp-ala is not affected by truncation of CTD

**Figure 7:**
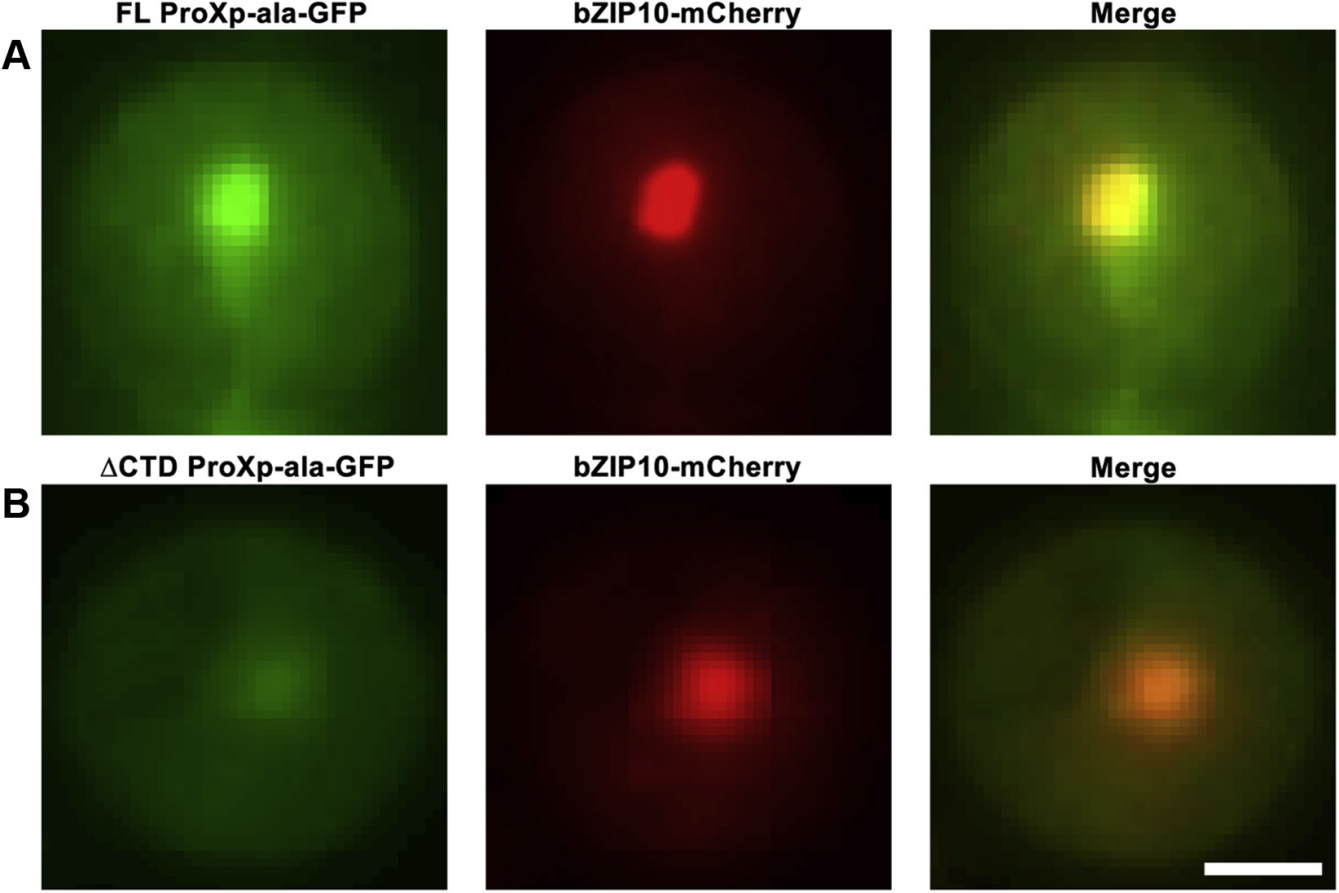
**Subcellular localization of GFP-tagged ProXp-ala and mCherry-tagged bZIP10 in *A. thaliana* protoplasts visualized by fluorescence microscopy.** Nuclear-localized bZIP10-mCherry was colocalized with FL (*upper panels*, *A*) or ΔCTD (*lower panels*, *B*) ProXp-ala-GFP. The scale bar represents 10 μm.

The computational and *in vitro* studies suggest a role for the CTD in protein dimerization and tRNA binding. To explore the functional significance of the unique CTD of ProXp-ala in plants, we first investigated the role of the CTD in subcellular localization. Protoplasts from WT *At* plants were transformed with various *ProXp-ala* constructs and control genes. As a control, *At* protoplasts were transformed with a GFP gene under a constitutive promoter; the GFP protein exhibited expression throughout the cytoplasm and nuclei as expected (Fig. S2*A*). Protoplasts transformed with *full-length (FL) ProXp-ala-GFP* also showed expression in both the cytoplasm and nuclei (Fig. 7*A*). To confirm nuclear localization, we cotransformed the nuclear marker construct *bZIP10-mCherry* (42), together with *FL ProXp-ala-GFP*. As expected, bZIP10-mCherry was expressed exclusively in nuclei. The merged image supported the nuclear localization of FL ProXp-ala-GFP (Fig. 7*A*).

To determine if CTD affected the subcellular localization of ProXp-ala, we performed a localization analysis using a CTD truncated (ΔCTD) ProXp-ala-GFP fusion protein. Protoplasts transformed with *ΔCTD ProXp-ala-GFP* showed an expression pattern in both cytoplasm and nuclei similar to that of FL ProXp-ala-GFP (Fig. 7*B*). The merged images of ΔCTD ProXp-ala-GFP and bZIP10-mCherry indicated that truncation of the CTD of ProXp-ala did not alter its subcellular localization pattern (Fig. 7*B*).

### CTD of ProXp-ala confers homodimerization *in vivo*

**Figure 8:**
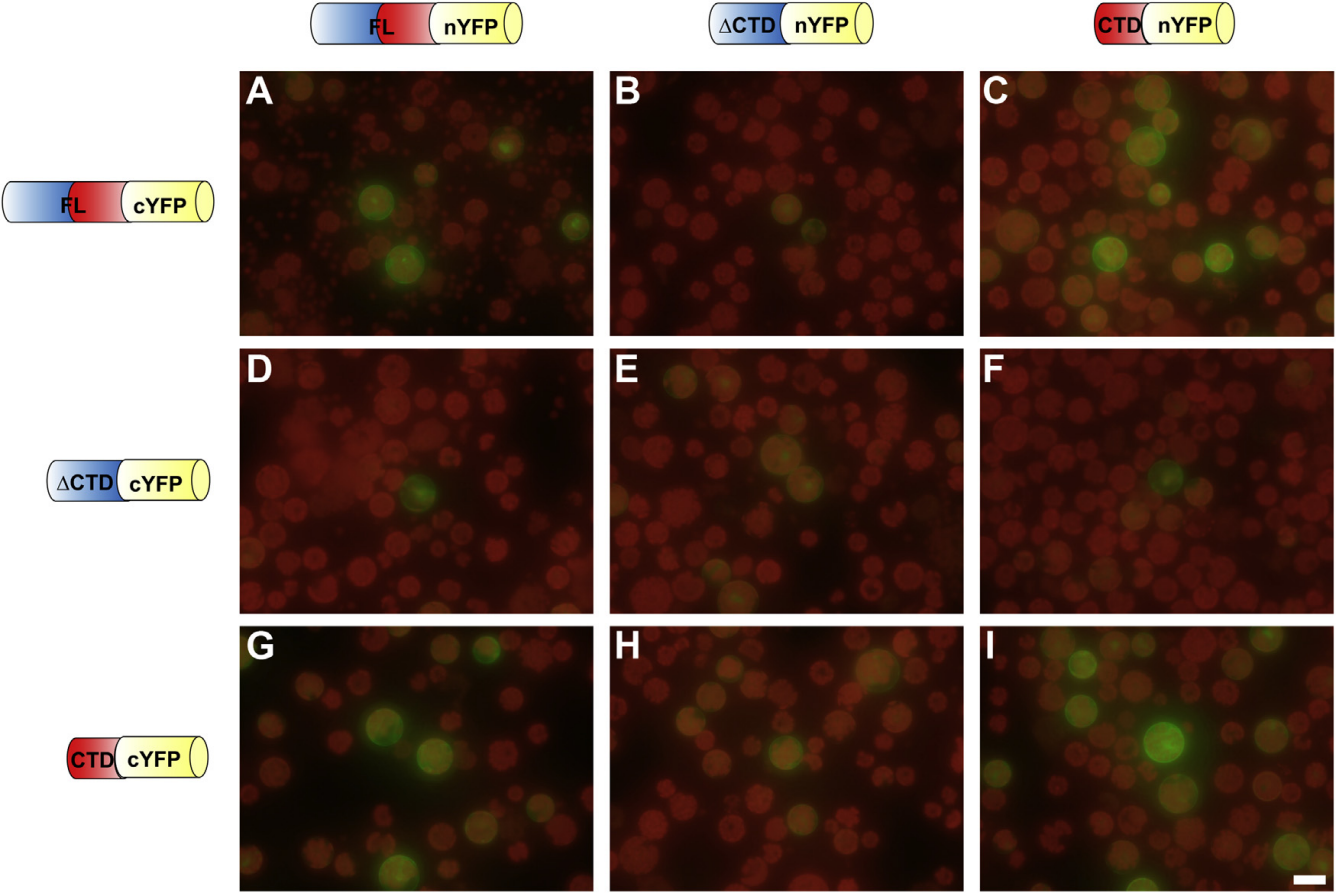
**Bimolecular fluorescence complementation analysis to determine the interaction between WT full-length (FL), ΔCTD, and CTD *At* ProXp-ala using an *A. thaliana* protoplast transient expression system.** N- and C-terminal split-YFP constructs were fused to the C terminus of FL, ΔCTD, and CTD ProXp-ala. The following nine combinations of constructs were coexpressed in *A. thaliana* protoplasts: (*A*) FL-nYFP and FL-cYFP, (*B*) ΔCTD-nYFP and FL-cYFP, (*C*) CTD-nYFP and FL-cYFP, (*D*) FL-nYFP and ΔCTD-cYFP, (*E*) ΔCTD-nYFP and ΔCTD-cYFP, (*F*) CTD-nYFP and ΔCTD-cYFP, (*G*) FL-nYFP and CTD-cYFP, (*H*) ΔCTD-nYFP and CTD-cYFP, and (*I*) CTD-nYFP and CTD-cYFP. Reconstituted YFP signals in the cytoplasm and nucleus indicate protein–protein interactions. The *red* background of the protoplasts is due to the autofluorescence of chloroplasts under UV light. The scale bar represents 20 μm.

Based on the predicted homodimerization function of the ProXp-ala CTD *in vitro*, a BiFC analysis was used to test the self-interaction of ProXp-ala in *At* protoplasts. A pair of split-YFP constructs, nYFP and cYFP, were each fused to the C termini of FL, ΔCTD, and CTD ProXp-ala. Thus, nine combinations of potential dimerization partners were tested (Fig. 8). The reconstitution of YFP fluorescence was observed upon coexpression of nYFP and cYFP-tagged FL ProXp-ala in both the nucleus and cytoplasm, supporting the homodimerization observed in the *in vitro* experiments (Fig. 8*A*). Notably, only weak YFP fluorescence was detected when ΔCTD ProXp-ala-nYFP or ΔCTD ProXp-ala-cYFP were coexpressed with the other pairs of split-YFP constructs, including FL, ΔCTD, and CTD ProXp-ala (Fig. 8*B*, *D–F*, *H*). We observed strong YFP signal in any combination where FL and CTD ProXp-ala were coexpressed (Fig. 8, *C* and *G*), while the pair of CTD constructs generated the strongest YFP signal (Fig. 8*I*). These data support a role for CTD in enhancing homodimerization of ProXp-ala.

To assess the specificity of the observed self-interaction of *At* ProXp-ala, various control experiments were also carried out. Expression of GFP alone demonstrated the high efficiency of protoplast transformation (Fig. S2*A*). bZIP25-cYFP protein was coexpressed with FL, ΔCTD, and CTD ProXp-ala-nYFP. As expected, no YFP fluorescence was detected upon coexpression of FL and CTD ProXp-ala with bZIP25, but weak YFP signals were detected upon coexpression of ΔCTD ProXp-ala with bZIP25. As a positive control, a pair of known interacting partners, bZIP1 and bZIP25, were coexpressed in *At* protoplasts and YFP fluorescence signal was detected in the nucleus, as expected (43) (Fig. S2*B*).

**Figure 9:**
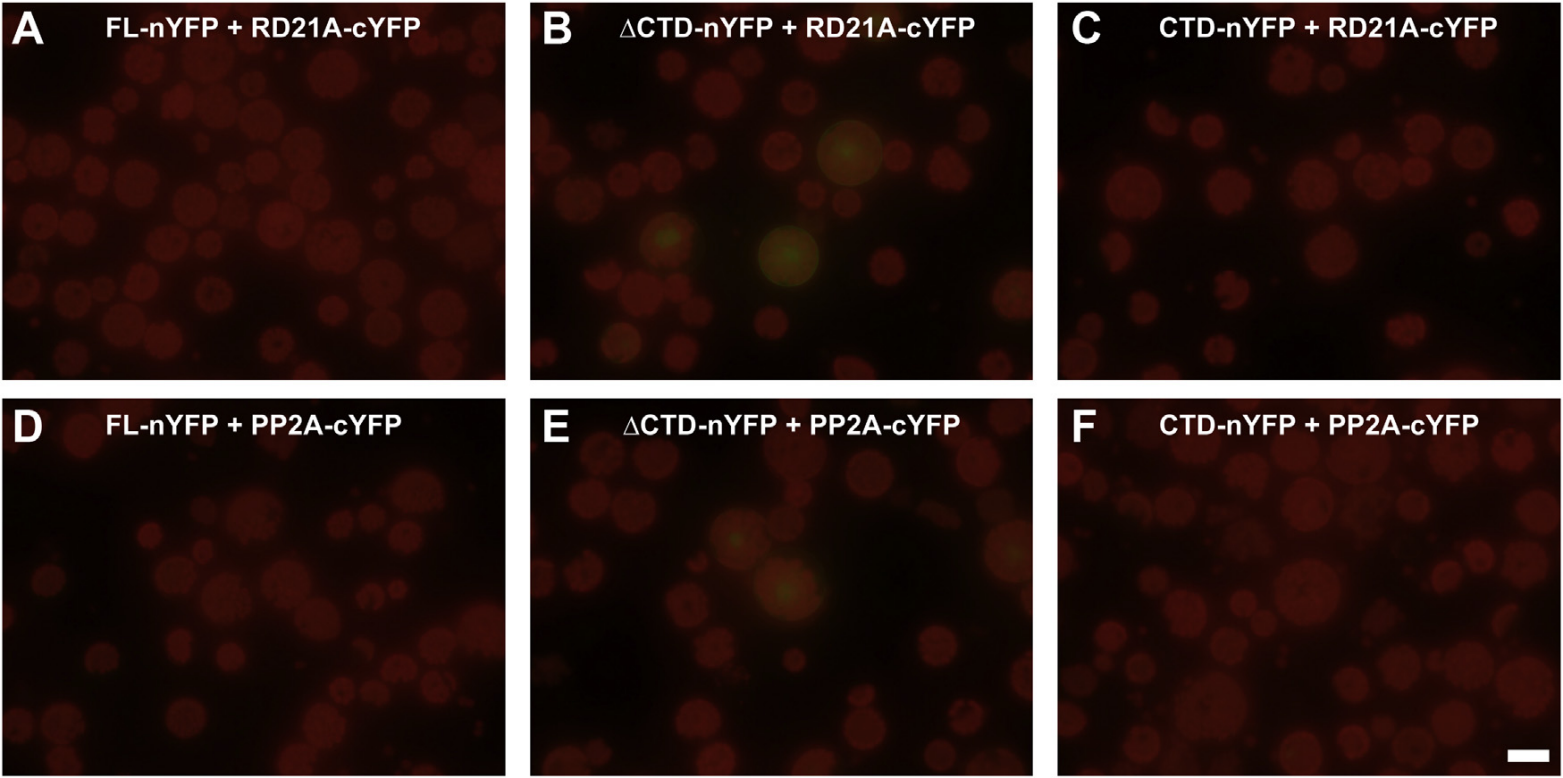
**Bimolecular fluorescence complementation analysis to probe the interaction between ProXp-ala and two unrelated proteins using an *A. thaliana* protoplast transient expression system.** A split nYFP domain was fused to the C terminus of FL, ΔCTD, and CTD ProXp-ala, while a split cYFP domain was fused to a cysteine protease (RD21A) and an isoform of protein phosphatase 2A (PP2A). (*A*–*C*) Coexpression of FL-, ΔCTD-, and CTD-nYFP with RD21A-cYFP. (*D*–*F*) Coexpression of FL-, ΔCTD-, and CTD-nYFP with PP2A-cYFP. Protein–protein interactions are indicated by a reconstituted YFP signal. The scale bar represents 20 μm.

To further confirm the specificity of ProXp-ala self-interaction in cells, we performed BiFC analysis using two unrelated proteins previously unknown to interact with ProXp-ala, RD21, and PP2A (44). FL and CTD ProXp-ala coexpression with RD21A and PP2A did not produce YFP fluorescence signals (Fig. 9*A*, *C* and *D*, *F*). However, when ΔCTD ProXp-ala was coexpressed with these proteins, weak YFP fluorescence signals were detected in the nuclei (Fig. 9, *B* and *E*). This result suggested that *At* ProXp-ala without the CTD exhibits weak nonspecific binding to unrelated proteins.

## Discussion

Over the course of evolution, aaRSs acquired additional domains and insertions to their catalytic cores as selective evolutionary adaptations to enhance their specificity through new molecular interactions and to stabilize the multidomain architecture of extant aaRSs (45, 46, 47). Specific examples of functionally important domains inserted into some bacterial ProRSs include the INS domain that improves fidelity by editing mischarged Ala-tRNA^Pro^ and the anticodon binding domain that improves the selectivity of specific tRNA^Pro^ substrates (17). Many eukaryotic aaRSs have evolved protein-binding domains unrelated to aminoacylation and editing functions, and these later additions to aaRSs correlate with the complexity of eukaryotic evolution (48).

Novel domains in protein-coding genes emerge during evolution through various mechanisms including genome insertion, deletion, rearrangement, and duplication; divergence from ancestral coding sequences; and *de novo* generation from previously noncoding DNA (49). These unique elements may result in the addition of new structures and functions to proteins that have been previously characterized in other organisms. Owing to their sessile and photosynthetic nature, the evolution of protein domain architecture is particularly necessary in plants. A recent genome-wide bioinformatics analysis in plants identified 59 aaRS-related genes, one of the largest sets of aaRS genes found in any organism (33). Some genes lacked canonical aaRS domains, whereas others encoded “extra” domains of unknown function. For example, one plant cytosolic histidyl-tRNA synthetase gene encodes a 400-residue-long N-terminal extension with unknown function that is unique to plant histidyl-tRNA synthetase (33). It has been suggested that these unique plant-specific domains may have noncanonical functions (33).

In this work, we demonstrated that plant ProXp-ala displays robust Ala-tRNA^Pro^ editing activity *in vitro* and showed that the unique CTD encoded in all plant ProXp-ala genes plays an important role in tRNA binding *in vitro*, as well as homodimerization both *in vitro* and *in vivo*. The lack of significant primary sequence conservation in the CTD among different plant species, despite high predicted secondary structure conservation, suggests that its evolutionary retention in plants is based on structure rather than on genome lineage. All three proteins with sequence homology to the plant CTD sequences—two independent plant proteins and one bacterial antigen—encode an α-helical structure. Given that independent intact genes or domains homologous to the plant CTD could not be identified in any plant genomes, the possibility that the CTD was appended to ProXp-ala *via* recombination is not substantiated. In addition, the *K*_d_ of the human Ala-tRNA^Pro^–human ProXp-ala interaction was previously determined to be 10.2 μM (29), a value very similar to that of ΔCTD *At* ProXp-ala binding to *At* Ala-tRNA^Pro^ reported in this work. Since the human enzyme lacks a CTD, these data suggest the presence of a common ancestral eukaryotic ProXp-ala gene that adopted a CTD in plants around the time they diverged from animals.

The CTD also confers protein homodimerization capability to *At* ProXp-ala, a characteristic that has not been observed in other INS superfamily members. Whether this domain also expands the plant enzyme’s protein–protein interactions to other binding partners is an open question. A recent study characterizing protein complexes in plants identified *At* ProXp-ala to be one of several proteins associated with a plant multi-aminoacyl-tRNA synthetase complex (MSC) (50). MSCs have previously been identified in yeast, mammals, and trypanosomes (50, 51). In humans, the architecture of the MSC is stabilized by several essential domains appended to aaRSs such as the glutathione s-transferase domains found in four components of the MSC (methionyl-tRNA synthetase, glutaminyl-prolyl-tRNA synthetase, AIMP2, and AIMP3) (52). Human ProXp-ala does not assemble with other components of the MSC. The observations that the *At* CTD mediates protein–protein interactions and that ProXp-ala participates in the MSC in plants suggest that the CTD may play a role in MSC assembly. While the present studies confirm a significant role of the plant CTD in homodimerization and substrate binding related to canonical editing function, future studies are needed to determine whether the CTD interacts with known plant MSC members and/or other cellular proteins in mediating noncanonical functions.

## Experimental procedures

### Sequence alignment and structural predictions

Bacterial, animal, and plant ProXp-ala sequences were retrieved from the BLAST searches of known bacterial INS-like domains and aligned using the Clustal Omega protein sequence alignment tool (53). Secondary structure and solvent accessibility predictions were performed using the primary sequence of *At* ProXp-ala as a query using the Porter, PaleAle 4.0 protein structure prediction tool (35). Structural modeling of *At* ProXp-ala was performed using the AlphaFold server (36, 37), and homo-oligomerization predictions were performed using the GalaxyWEB GalaxyHomomer *ab initio* homo-oligomer prediction tool (40).

### Protein preparation

The *At* *ProXp-ala* (*Arabidopsis* Genome Initiative locus code: AT1G44835) gene was synthesized and cloned into vector pET15b (Novagen) with an N-terminal His-tag and thrombin cleavage site using restriction sites NdeI and BamHI by GENEWIZ. Protein expression was carried out in *E. coli* BL21 CodonPlus (DE3) RIL cells (Agilent Technologies). DNA encoding ΔCTD was generated by removing residues 166 to 307 of WT *At* ProXp-ala *via* site-directed, ligase-independent mutagenesis (primers shown in Table S1) (54). DNA encoding only the CTD of *At* ProXp-ala (residues 166–307) with an N-terminal His-tag in pET15b was a gift from Dr Dominic Qualley (Berry College). All sequences were confirmed by DNA sequencing carried out by The Genomics Shared Resource at The Ohio State University Comprehensive Cancer Center. *E. coli* BL21-CodonPlus (DE3)-RIL (Agilent Technologies) cells were transformed with each plasmid and grown in Luria–Bertani broth to *A*_600_ = 0.4 to 0.8. Protein overexpression was carried out by induction with 100 μM isopropyl β-D-1-thiogalactopyranoside for 18 to 20 h at 20 °C. The cells were centrifuged at 6000*g* for 15 min at 4 °C and then lysed by 10 mg/ml lysozyme in lysis buffer (50 mM sodium phosphate pH 7.5, 600 mM NaCl, 10% v/v glycerol, 20 mM β-mercaptoethanol) with 1 cOmplete EDTA-free protease inhibitor tablet (Roche). The lysate was homogenized by nine rounds of sonication (1-s pulses for 20 s at level 5.0) followed by centrifugation at 27,000*g* for 30 min at 4 °C. The supernatant was collected and passed through a 0.45-μm filter before being loaded onto a His-Select Nickel affinity chromatography column (Sigma-Aldrich). A 5-ml stepwise imidazole gradient (20, 30, 40, 60, 80, and 250 mM) was used to elute each protein from the column. Each elution was analyzed by denaturing SDS-PAGE. For WT, ΔCTD, and CTD *At* ProXp-ala, the fractions containing 60, 80, and 250 mM imidazole were concentrated and exchanged into storage buffers using Amicon-Ultra spin concentrators (10k MWCO for WT and 3k MWCO for ΔCTD and CTD). The final storage buffer conditions were optimized for WT and CTD (50 mM sodium phosphate pH 6.0, 500 mM NaCl, and 1 mM DTT) and for ΔCTD (50 mM sodium phosphate pH 7.5, 150 mM NaCl, and 1 mM DTT). Concentrated proteins were mixed 1:1 v/v with 80% glycerol and stored at −20 °C. Enzyme concentrations were determined using a Bio-Rad Protein Assay Kit using bovine serum albumin as a standard.

### tRNA and aminoacyl-tRNA substrate preparation

The Genomic tRNA Database (gtRNAdb) was used to identify the most abundant isoacceptor sequence of tRNA^Pro^ in the *At* genome (55). The sequence of *At* tRNA^Pro(UGG)^ preceded by the T7 RNA polymerase promoter sequence (Fig. S3) was synthesized and cloned into vector pUC57 by GENEWIZ. The T7 promoter-tRNA gene region was amplified by PCR (primers shown in Table S2) and *in vitro* transcribed with recombinantly expressed and purified T7 RNA polymerase (56). Transcribed tRNAs were purified by denaturing 12% urea polyacrylamide gel electrophoresis followed by excision of the tRNA band. The gel pieces were crushed and soaked in RNA elution buffer (500 mM ammonium acetate and 1 mM EDTA) at 37 °C overnight. The eluent was passed through a 0.45-μm filter before being concentrated *via* extraction with butanol. The concentrated tRNAs were ethanol precipitated and resuspended in RNase-free Millipore water. Concentrations of tRNA stocks were determined by measuring the UV absorbance at 260 nm and using an extinction coefficient of 0.604 μM^−1^ cm^−1^.

Prior to use in deacylation assays, tRNAs were 3′-end radiolabeled with ^32^P using [^32^P]-ATP (PerkinElmer) and *E. coli* tRNA nucleotidyltransferase, prepared as described (57). Aminoacylated tRNA^Pro^ substrates were generated by addition of 10 μM cold tRNA^Pro^ to ethanol-precipitated [^32^P]-tRNA^Pro^ and incubating the tRNA mixture with 10 μM *At* ProRS, 4 mM ATP, 0.03 mg/ml pyrophosphatase and either 900 mM alanine or 30 mM proline in aminoacylation buffer (50 mM Hepes, pH 7.5, 20 mM KCl, 20 mM β-ME, 10 mM MgCl_2_, 0.1 mg/ml bovine serum albumin) for 5 min at 37 °C. Aminoacyl-tRNAs were recovered by phenol chloroform extraction followed by ethanol precipitation, then stored in 3 mM sodium acetate pH 5.2 at −80 °C. The concentration of aminoacyl-tRNAs was determined by comparing the radioactive signal of charged tRNA with that of a 5 μM uncharged standard after digestion with S1 nuclease (final concentrations: 4 U/μl S1 nuclease and 100 mM sodium acetate pH 5.0 in S1 nuclease buffer [Promega]) and spotting on a polyethylenimine-cellulose thin layer chromatography (TLC) plate (EMD Millipore) using a mobile phase of 0.05% ammonium chloride and 5% acetic acid by volume (57). Radioactive species were detected by phosphorimaging using a Typhoon FLA 9500 instrument and quantified using ImageQuant TL 8 software.

### Deacylation assays

Single-turnover aa-tRNA deacylation reactions were performed as described (26, 57) using 10 nM aa-tRNA and 500 nM enzyme in deacylation buffer (50 mM Hepes pH 7.0, 20 mM KCl, 5 mM MgCl_2_, 0.1 mg/ml bovine serum albumin, and 2 mM DTT) at 25 °C unless otherwise noted. At each indicated time point, 2-μl aliquots of the reaction were mixed with 6 μl of S1 quenching solution as described above. Product formation was monitored by separating aa-[^32^P]-AMP from uncharged [^32^P]-AMP on PEI-cellulose TLC, and radioactive species were detected as described above. Observed rate constants, *k*_obs_, were obtained by fitting the time course of aa-tRNA deacylation to the single-exponential equation y = y_0_ e^-k^_obs_^t^ using Prism 9 (GraphPad Software). Each reported rate constant is the average of at least three independent assays, and the amount of remaining aa-tRNA at each timepoint was corrected for nonenzymatic buffer hydrolysis.

For *K*_M(STO)_ determination under STO conditions, deacylation reactions were performed with varying enzyme concentrations using a range of 0 to 1 μM WT *At* ProXp-ala and 0 to 20 μM *Δ*CTD *At* ProXp-ala. Using Prism 9, the *K*_M(STO)_ and *k*_obs,max_ values were obtained by fitting the *k*_obs_ *versus* [enzyme] plot with the standard Michaelis–Menten equation for ΔCTD: *k*_obs_ = *k*_obs,max_ [ΔCTD]/*K*_M(STO)_ + [ΔCTD]) or the allosteric sigmoidal equation for WT: *k*_obs_ = *k*_obs,max_ [WT]^h^/(*K*_M(STO)_^h^ + [WT]^h^), where h is a measure of cooperativity called the Hill slope.

Pulse-chase experiments to establish that *k*_off_ >> *k*_cat_ were performed as described (58, 59). Briefly, a deacylation assay was initiated using 100 nM *At* Ala-[^32^P]-tRNA^Pro^ and 750 nM WT *At* ProXp-ala and the reaction progress was monitored as described above. After 60 s, the reaction was pulsed with a 100-fold dilution of reaction buffer containing 1.0 μM uncharged, unlabeled *At* tRNA^Pro^. Aliquots were withdrawn as described above for the remainder of the time course. Control reactions were performed (a) without the pulse at 60 s and (b) by initiating the reaction with the 100-fold competitive dilution. Each data point is the average of three independent trials with error bars indicating the standard deviation. Trendlines were obtained by fitting each time course to a single-exponential decay equation.

### CD spectroscopy and SEC-MALS

CD spectra were recorded on a Jasco J-815 CD Spectrometer using 0.5 mg/ml WT, ΔCTD, or CTD *At* ProXp-ala in 50 mM sodium phosphate pH 5.6 (WT and CTD) or pH 7.5 (ΔCTD). Raw CD signal in millidegrees was converted to molar ellipticity and plotted against wavelength in nanometers. For SEC-MALS analyses, 100 μl solutions of 3 mg/ml WT, 6 mg/ml ΔCTD, or 1.5 mg/ml CTD *At* ProXp-ala were injected onto a Superdex 200 Increase 10/300 GL size-exclusion column equilibrated in MALS buffer (50 mM Hepes pH 6.8, 30 mM KCl, 2 mM DTT, and 1 mM MgCl_2_). MALS analysis was performed with a DAWN HELIOS 8+ laser photometer and an Optilab T-rEX differential refractometer (Wyatt Technology), which measure the light scattering intensity and differential refractive index of the column eluate, respectively. ASTRA 7.1.4 software was used to align the UV, light scattering, and differential refractive index signals, before fitting the light scattering data to a Zimm model, which was used to determine the weight average molecular mass of particles within a given light scattering peak (60).

### Plant material and growth conditions

All *A. thaliana* plants used in this study were Columbia-0 (Col-0) plants grown under 16-h-light/8-h-dark cycles at 20 °C to 22 °C in growth chambers at the Biotechnology facility at OSU. Isolation and transient gene expression of protoplasts and *A. thaliana* plant transformation were carried out as described (61, 62, 63).

### Subcellular localization and bimolecular fluorescence complementation

Molecular cloning for subcellular localization analysis was performed using Gateway (Invitrogen) technologies according to manufacturer’s instructions. All DNA primers used are reported in Table S3. PCR-amplified cDNAs encoding FL and ΔCTD ProXp-ala were inserted into pENTR/D-TOPO vectors and then transferred to a modified Gateway-compatible pBluescript KS+ destination vector, which contains GFP fused in-frame at the C terminus of the inserted gene (61). A vector encoding the basic leucine zipper 10 protein (bZIP10) fused with mCherry was cotransformed into *At* protoplasts with the GFP-tagged ProXp-ala constructs and served as a nuclear marker. The strong and constitutive CaMV35S-GFP reporter construct was transformed in parallel for each experiment to serve as an indicator of protoplast transformation efficiency.

For the BiFC analysis, cDNAs encoding FL, ΔCTD, and CTD ProXp-ala without stop codons were cloned into split-YFP vectors, pA7-nYFP and pA7-cYFP, under the control of the *CaMV35S* promoter (64, 65). All DNA primers used for BiFC are reported in Table S4. The pair-wise constructs were cotransformed into protoplasts isolated from leaves of WT Col-0 *At* plants as described (64). The *CaMV35S-GFP* construct was used again as a control for transformation efficiency. Additional controls that were tested are split-YFP vectors encoding cysteine proteinase (RD21A) and one isoform of protein phosphatase 2A (PP2A).

### Fluorescence microscopy

Fluorescence microscopy was carried out using a Nikon Eclipse E600 fluorescence microscope with appropriate filter sets (Nikon Instruments Inc) to detect subcellular localization patterns of GFP-fusion proteins and YFP signals in BiFC assays. GFP and YFP fluorescence was detected using an excitation filter of 450 to 490 nm, a dichroic mirror of 500 nm, and a barrier filter of 515 nm, while mCherry fluorescence was visualized using an excitation filter of 540 to 580 nm, a dichroic mirror of 595 nm, and a barrier filter of 600 to 660 nm. A 1-s exposure time was used for all experiments. Images were captured by a SPOT RT Slider multimode camera and Advance SPOT Software 5.0 (Diagnostics Instruments). Protoplast samples (9–10 μl) were loaded onto a Bright-Line hemacytometer for microscope observation.

## Data availability

All data described are contained within the article.

## Supporting information

This article contains supporting information (25, 53, 55, 66).

## Conflict of interest

The authors declare that they have no conflicts of interest with the contents of this article.

## Supporting information

2022_05_23_JBC_At ProXp-ala_Supporting Information_FINAL
