# Supplementary material for: Plant-exclusive domain of trans-editing enzyme ProXp-ala confers dimerization and enhanced tRNA binding
Source: J Biol Chem. 2022 Jul 12;298(9):102255. doi: 10.1016/j.jbc.2022.102255 (PMC9425024; doi:10.1016/j.jbc.2022.102255)
Supplement: 2022_05_23_JBC_At ProXp-ala_Supporting Information_FINAL [file mmc1.docx]

Supporting information for

**Plant-exclusive domain of *trans*-editing enzyme ProXp-ala confers dimerization and enhanced tRNA binding**

Jun-Kyu Byun^1,2 †^, John A. Vu^1,2 †^, Siou-Luan He^1,3^, Jyan-Chyun Jang^1,3*^, and Karin Musier-Forsyth^1,2*^

^1^Center for RNA Biology, The Ohio State University, Columbus, OH 43210, USA.

^2^Department of Chemistry and Biochemistry, The Ohio State University, Columbus, OH 43210, USA.

^3^Department of Horticulture and Crop Science and Center for Applied Plant Sciences, The Ohio State University, Columbus, OH 43210, USA.

**Supporting Information**

Figures S1, S2, and S3

Table S1, S2, S3, and S4


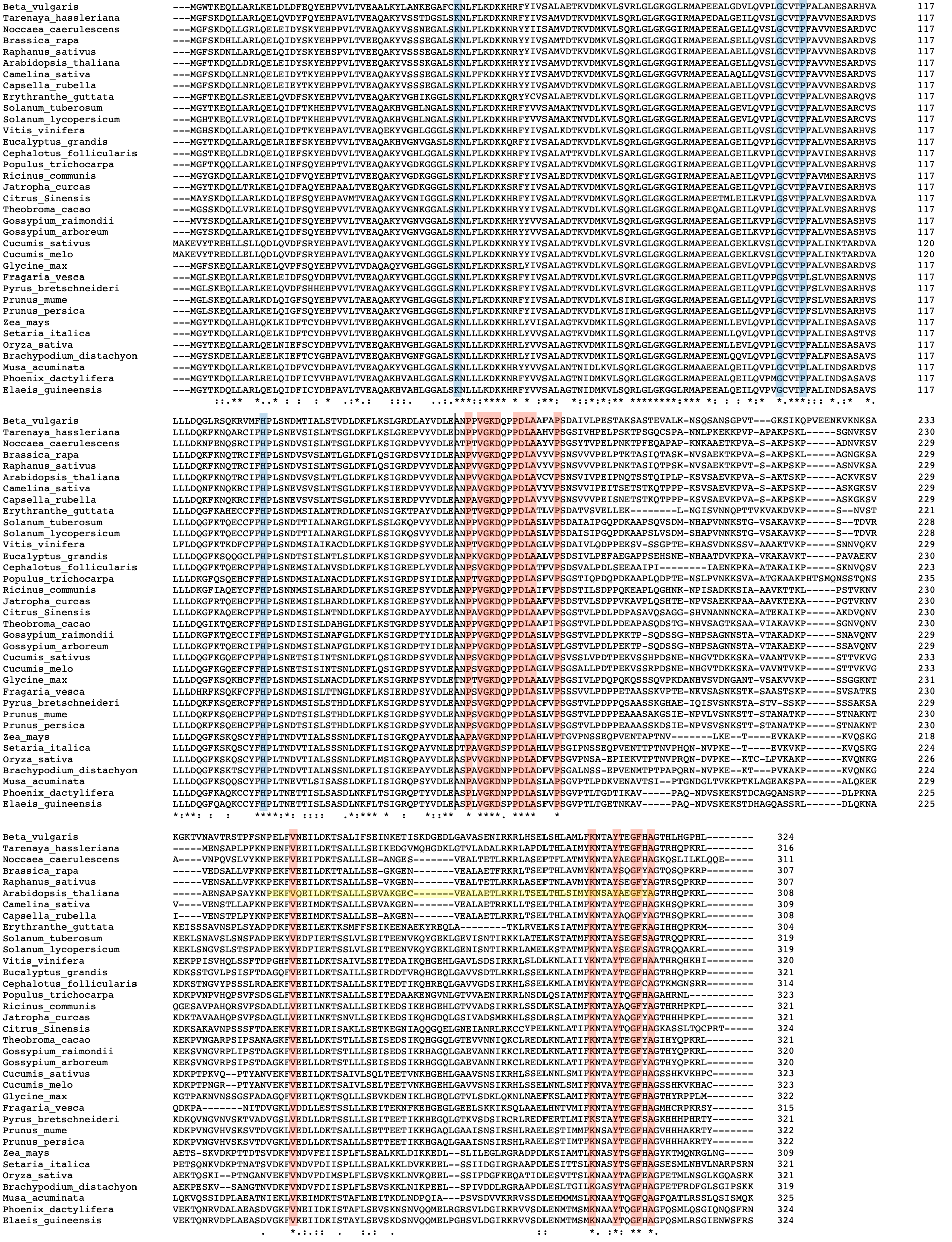


**Figure S1. Multiple sequence alignment of ProXp-ala from 35 plant species.** Asterisks (*) indicate positions with perfectly conserved residues, colons (:) indicate positions with conserved residues with strongly similar properties, and periods (.) indicate positions with conserved residues with weakly similar properties (53). Conserved residues in the N-terminal ProXp-ala catalytic core are highlighted in blue and include the strictly conserved Lys residue of INS-like editing domains, the GXXXP loop, and the gatekeeper histidine (25,66). A black vertical line indicates the start of the plant-exclusive CTD. Strictly conserved residues within the plant CTD are highlighted in red. Helical residues proposed to make up the homodimerization motif of *At* ProXp-ala are highlighted in yellow.


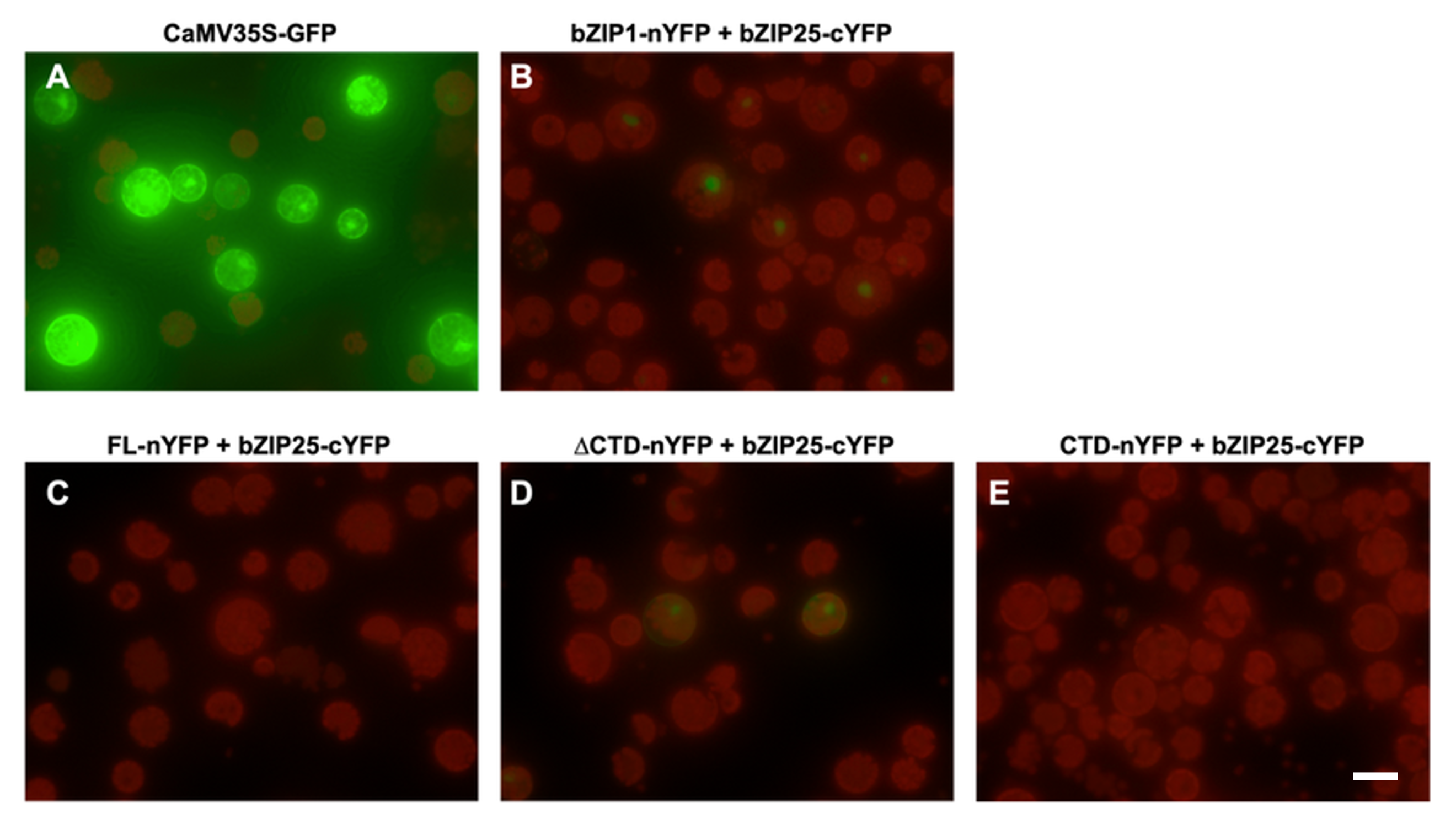


**Figure S2. BiFC analysis to determine the interaction between bZIPs and various fragments of ProXp-ala using an *A. thaliana* protoplast transient expression system.** (A) CaMV35S-GFP was used as a control for protoplast transformation efficiency. (B) bZIP1-nYFP and bZIP25-cYFP were co-expressed as a positive control pairing known to interact in the nucleus. (C-E) bZIP25-cYFP was co-expressed with FL, ∆CTD, and CTD ProXp-ala fused with nYFP, as indicated. Protein-protein interactions are indicated by the reconstituted YFP signal. Note that ∆CTD ProXp-ala interacts weakly with bZIP25 in the nucleus (D). Scale Bar, 20 µm.


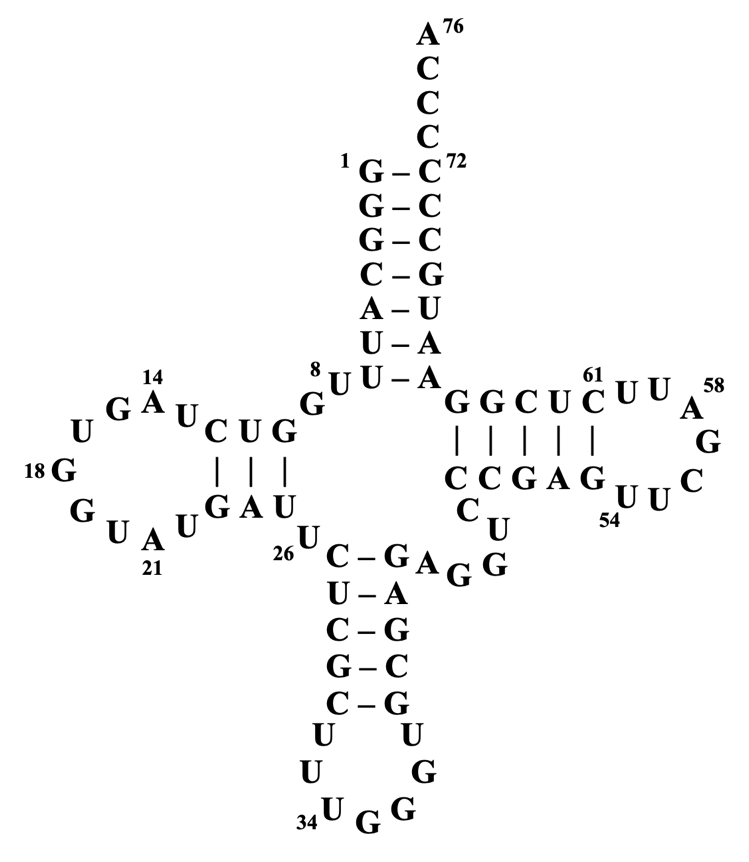


**Figure S3. Sequence and secondary structure of *At* tRNA^Pro^ used in this work.** Sequence of the most abundant isoacceptor (UGG) according to the Genomic tRNA Database is shown (gtRNAdb) (55).

**Table S1. SLIM Primers for deletion of ProXp-ala CTD**

| **Purpose** | **Use** | **Sequence** |
| --- | --- | --- |
| Protein expression of ΔCTD | Short Forward | 5′ TAAGGATCCGGCTGCTAAC 3′ |
|  | Long Forward | 5′ CGTATACGTTGACCTTGAGTAAGGATCCGGCTGCTAAC 3′ |
|  | Short Reverse | 5′ GGATCTCTCCCAATCGACTTC 3′ |
|  | Long Reverse | 5′ CTCAAGGTCAACGTATACGGGATCTCTCCCAATCGACTTC 3′ |

**Table S2. Primers of tRNA^Pro(UGG)^**

| **Purpose** | **Use** | **Sequence** |
| --- | --- | --- |
| PCR amplification of tRNA^Pro(UGG)^ | Forward | 5′ CGGATAACAATTTCACACAG 3′ |
|  | Reverse | 5′ TGGGGGGCATTCCGAGAATC 3′ |

**Table S3. Primers used for sub-localization assay**

| **Purpose** | **Primers** | **Sequence** |
| --- | --- | --- |
| Protein expression of FL and ΔCTD | Forward | 5′ CACCATGGGTTTCACCAAAGATCAG 3′ |
| Protein expression of FL | Reverse | 5′ CAATCGCTTCGGCTGGTGG 3′ |
| Protein expression of ΔCTD | Reverse | 5′ CTCAAGGTCAACGTATACGG 3′ |

**Table S4. Primers used for BiFC**

| **Purpose** | **Primers** | **Sequence** |
| --- | --- | --- |
| Protein expression of FL and ΔCTD | Forward | 5′ CCGCTCGAGATGGGTTTCACCAAAGATCAG 3′ |
| Protein expression of CTD | Forward | 5′ CCGCTCGAGATGGCTAACCCGGTAGTTGGTAAAG 3′ |
| Protein expression of FL and CTD | Reverse | 5′ GGACTAGTCAATCGCTTCGGCTGGTGG 3′ |
| Protein expression of ΔCTD | Reverse | 5′ GGACTAGTCTCAAGGTCAACGTATACGG 3′ |
